# Comparison of short-term efficacy of MIS-TLIF and Endo-LIF in the treatment of single-segment degenerative lumbar diseases

**DOI:** 10.3389/fsurg.2022.922930

**Published:** 2022-09-23

**Authors:** Zhiwei Song, Weihua Zhu, Junwen Zheng, Gang Wu, Tianqi Li, Aibing Huang, Jian Bian, Chunmao Chen, Haijun Li

**Affiliations:** ^1^ Department of Orthopedics, Hospital Affiliated 5 to Nantong University, Taizhou People’s Hospital, Taizhou, China; ^2^ Medical School of Nantong University, Nantong, China; ^3^ Department of Orthopedics, Taizhou People’s Hospital, Nanjing Medical University, Taizhou, China; ^4^ Postgraduate Training Base of Dalian Medical University, Taizhou People’s Hospital, Jiangsu, China

**Keywords:** lumbar degenerative disease, minimally invasive surgery, lumbar vertebrae, minimally invasive, pedicle screw

## Abstract

**Background:**

A prospective controlled study was conducted to compare the short-term clinical results and postoperative complications of minimally invasive transforaminal lumbar decompression and fusion (minimally invasive surgery transforaminal lumbar interbody fusion, MIS-TLIF) and percutaneous endoscope-assisted transforaminal lumbar interbody fusion (endoscopic lumbar interbody fusion, Endo-LIF) in the treatment of single-segment degenerative lumbar diseases, to provide some scientific guidance for clinicians to select surgical treatment for patients with lumbar degeneration.

**Methods:**

From October 2020 to October 2021, a total of 62 patients were enrolled, with 31 patients in the MIS-TLIF group and 31 patients in the Endo-LIF group. All patients were followed up for 6 months. The following information from the two groups of patients was recorded: (1) operation time, radiation exposure time, intraoperative blood loss, bed rest time, and hospital stay; (2) ODI score (The Oswestry Disability Index), low back pain VAS score (Visual Analogue Scale), and lumbar vertebra JOA score (Japanese Orthopaedic Association Scores) 1 day before the operation; 1, 3, 6 days after operation; and 1, 3 and 6 months after operation. (3) X-ray evaluations of lumbar fusion at the last follow-up.

**Results:**

There were significant differences in operation time, intraoperative fluoroscopy time, and hospitalization cost between the two groups. The MIS-TLIF group was significantly better than the Endo-LIF group, and the intraoperative bleeding volume of the Endo-LIF group was significantly better than that of the MIS-TLIF group, but there was no significant difference in postoperative bed rest time and postoperative hospital stay. There was no significant difference in the scores of ODI, VAS, and JOA between the two groups before and after the operation. At the last follow-up, the fusion rate was 100% in the MIS-TLIF group and 100% in the Endo-LIF group.

**Conclusions:**

There was no significant difference in short-term clinical efficacy and safety between Endo-LIF and MIS-TLIF in the treatment of single-segment degenerative lumbar diseases, but MIS-TLIF was significantly better than Endo-LIF in terms of the operation time, hospitalization cost, and fluoroscopy time, and Endo-LIF was significantly better than MIS-TLIF in terms of intraoperative blood loss.

## Introduction

Due to the aging of the global population, the incidence of degenerative lumbar diseases such as lumbar disc herniation, lumbar spinal stenosis, lumbar instability, and lumbar spondylolisthesis is gradually increasing (1). Lumbar degenerative disease of the lumbar spine is a significant cause of disability and healthcare burden with a global incidence of 266 million individuals or 3.63% of the world population (2). More than 30% of men and more than 40% of women suffer from low back pain every day (3). For patients who need surgery, lumbar interbody fusion (4, 5) is the main treatment choice. Over the past 10 years, great changes have taken place in the medical model. We have moved from traditional medicine to a minimally invasive surgical era. The concept has promoted the change in spinal surgery technology. Throughout the 100-year history of spinal surgery, spinal fusion technology runs through the history of spinal surgery. After development, anterior vertebral fusion (6, 7) in the 1930s, PLIF fusion in the 1950s, transforaminal lumbar interbody fusion (TLIF) fusion (8, 9) in the 1980s, the first minimally invasive surgery (MIS-PLIF) in 2002, to the first MIS-TLIF in 2006 (10).

In recent years, minimally invasive transforaminal lumbar decompression and fusion (MIS-TLIF) has been gradually applied to patients with lumbar degenerative diseases (11, 12). Percutaneous endoscope-assisted transforaminal lumbar decompression and fusion (Endo-LIF) (13) is a perfect combination of endoscopic decompression and fusion, and it is also a minimally invasive technique (14), but there are still few studies on its indications and clinical effects and there are few reports on its comparison with MIS-TLIF.

In this study, patients with lumbar degeneration were divided into two groups: the MIS-TLIF group and the Endo-LIF group. The perioperative data were observed to compare the clinical effects of the two surgical methods.

## Patients and methods

### General data of patients

Inclusion criteria (including general data of patients undergoing MIS-TLIF and Endo-LIF surgery, divided into groups according to random envelope method) were as follows: (1) the subjects were 70 years old (including 18 and 70 years old), regardless of gender; (2) the clinical symptoms were severe low back pain or low back pain, with or without intermittent claudication; (3) it was ineffective after strict conservative treatment for 3–6 months; (4) the imaging findings were single segmental spondylolisthesis (I–II degree), lumbar spinal stenosis, lumbar disc herniation, and consistent with clinical symptoms; (5) no mental illness, can tolerate surgery, and cooperate with followers. Exclusion criteria were as follows: (1) patients with multisegmental lumbar disease judged by symptoms, signs, and imaging examination; (2) patients with a history of mental illness; (3) patients with a history of lumbar surgery; (4) patients with severe osteoporosis (T value ≤ 2.5 measured by dual-energy x-ray absorption method); (5) patients with clear surgical contraindications, such as severe heart disease, diabetes, renal failure, respiratory failure, blood coagulation, and other serious medical diseases; (6) patients who refuse to sign the informed consent form.

Basic information of selected objects: According to the above criteria, 62 of 106 patients with lumbar degeneration met the inclusion criteria from October 2020 to October 2021, with 31 in the MIS-TLIF group and 31 in the Endo-LIF group (Table 1). This study was approved by the Ethics Committee of Taizhou People's Hospital. The clinical trial protocol number is ChiCTR2100043265. Prior to the study, all participants had signed a written informed consent form. Preoperative data collection included anterior and lateral lumbar radiographs, lateral flexion and extension films, lumbar MRI, and lumbar CT scans. The physical examination of all patients must be consistent with the results of the imaging examination. After the operation, the placement of the pedicle screw and interbody fusion cage was examined by routine x-ray and CT scan. A senior spinal surgeon (Li Haijun) has more than 15 years of experience in spinal surgery, with Li Haijun as the center, performing all surgical procedures.

**Table 1:** Demographic data for the patients in the two groups.

		Endo-LIF	MIS-TLIF	*P*
	Cases	31	31	—
	Gender (female/male)	17/14	11/20	—
	Age (years)	59.1 ± 8.77	54.81 ± 9.46	0.07
	BMI	22.14 ± 2.82	23.61 ± 3.07	0.06
Etiology	Disc herniation	17	24	0.20
	Lumbar spondylolisthesis (grade I–II)	7	3	—
	Lumbar spinal stenosis	4	1	—
	Single-level lumbar instability	3	3	—
Fusion levels	L3/4	1	-	0.77
	L4/5	24	22	—
	L5/S1	6	9	—

Endo-LIF, endoscopic lumbar interbody fusion; MIS-TLIF, minimally invasive surgery-transforaminal lumbar interbody fusion.

Preoperative preparation: x-ray, CT, MRI, and other imaging examinations of lumbar vertebrae were perfected before the operation to confirm the diagnosis. At the same time, improve the routine examination of electrocardiogram, urine routine, blood routine, stool routine, chest CT scan, electrolyte, blood coagulation function, liver and kidney function, hepatobiliary pancreas and spleen ultrasound, lower extremity vein ultrasound, heart color ultrasound, and so on. If the patient has mild medical disease, the corresponding symptomatic support treatment will be given first, and the surgical treatment will be feasible after the abnormal physiological indexes are restored to the safe range allowed by the operation.

**Endo-LIF Group** (Take the L4/L5 segment as an example, the left approach).

**Figure 1:**
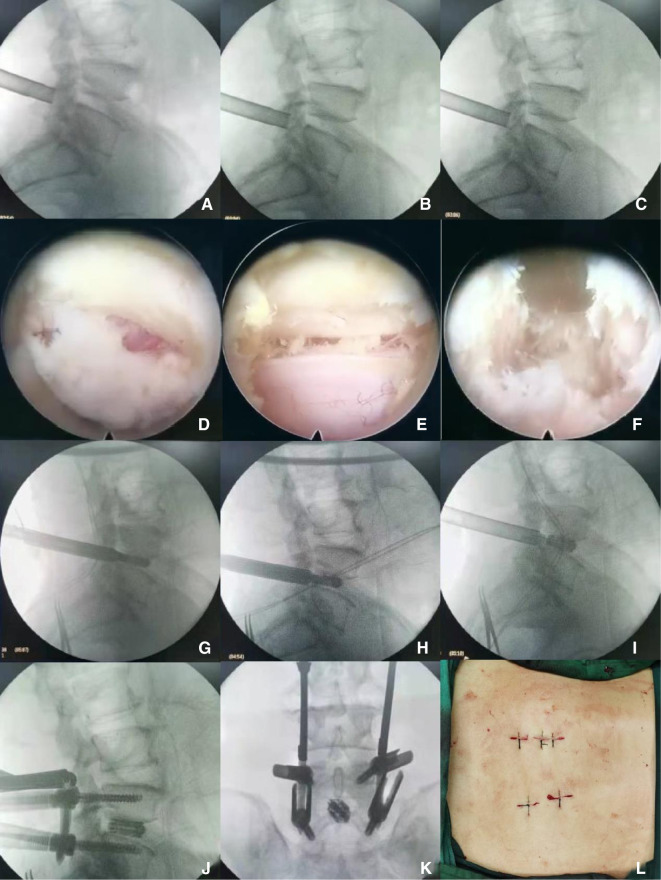
(**A–C**) show that the lumbar vertebrae of Endo-LIF operation were successfully punctured laterally, and the inferior articular process was removed by fluoroscopic saw ring; (**D–F**) were performed under Endo-LIF operation microscope; (**G–I**) were implanted with Endo-LIF fusion cage; (**J**) Endo-LIF lumbar percutaneous nailing lateral film; (**K**) Endo-LIF lumbar percutaneous nailing positive film; (**L**) Endo-LIF incision.

1. After the patient's general anesthesia was successful, take the prone position and disinfect the skin with 0.5% iodophor three times before spreading the sterile towel.
2. C-arm locates the left intervertebral space of lumbar 4 and 5, cuts the skin and subcutaneous tissue, deep fascia, puncture rod punctures the inferior articular process of the upper vertebral body, expands the cannula to the diameter of 10 mm, removes the articular process under fluoroscopy, chapter 2 materials and methods 4 the farthest end, access to the endoscopic channel, the ring saw cuts off the articular process and the base of the spinous process again, and the left half of the remaining lumbar 4 lamina is resected with gun forceps. From the proximal end to the attachment point of the ligamentum flavum, the bilateral ligamentum flavum was removed (through the unilateral channel, bilateral resection of the ligamentum flavum), the working sleeve was rotated, the spinal cord and nerve root were blocked to the opposite side, and the left intervertebral space was exposed. The intervertebral nucleus pulposus and cartilage endplate were removed with nucleus pulposus forceps and a scraper. Rinse the intervertebral space with normal saline, crush the articular process and lamina into the intervertebral space, and then insert the interbody fusion cage filled with bone block until the position is confirmed by fluoroscopy. Finally, the nerve root canal is explored and enlarged without compression.
3. Confirm and locate the “eye” position of L4 and L5 bilateral pedicles under C-arm fluoroscopy, and place the needle tip on the outer upper edge of the pedicle shadow. Under the monitoring of the C-arm machine, drill into the puncture needle, when the positive position shows that the puncture needle reaches the medial edge of the pedicle shadow, the lateral position shows that the needle tip reaches the posterior wall of the vertebral body, indicating that the safe puncture is completed, pull out the inner core of the puncture needle, and place the human guide needle. Four pedicle screws were implanted into the pedicle along the guide needle. Then, the guide needle was removed and the rod holder was implanted to connect the titanium rod and nut. Fluoroscopy showed that the reduction of L4 and L5 vertebrae was satisfactory.
4. After strictly counting gauze and instruments, alcohol disinfects the skin, and the wound is sutured with a single needle. (There were no difficulties in monosegmental L5-S1 disc herniations, in terms of surgical access with Endo-LIF in patients with high iliac bone position) (Figure 1).

**MIS-TLIF Group** (Take the L4/L5 segment as an example, the right approach).

**Figure 2:**
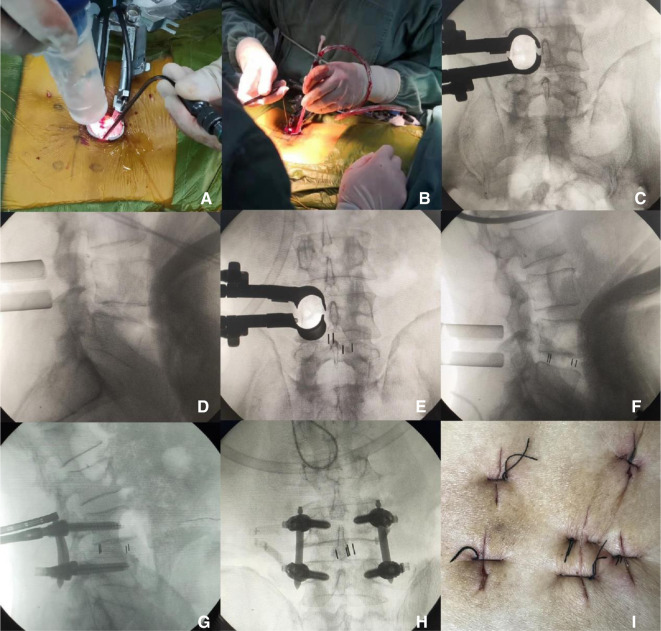
MIS-TLIF operation shown in (**A,B**); MIS-TLIF cage placement shown in (**C–F**); percutaneous nailing shown in MIS-TLIF in (**G,H**), and MIS-TLIF incision shown in (**I**).

1. After the patient's general anesthesia was successful, take the prone position and disinfect the skin with 0.5% iodophor three times before spreading the sterile towel.
2. C-arm locates the right intervertebral space of L4, L5, cuts the skin, subcutaneous tissue, and deep fascia layer by layer, punctures the puncture rod to the inferior articular process of the upper vertebral body, expands the cannula to the diameter of 14 mm, inserts the quadrant minimally invasive channel and expands the soft tissue, installs and connects the light source, removes the soft tissue that slips out of the muscle and the surface of the articular process, exposes the inferior articular process, and excises the inferior articular process with an ultrasonic knife or bone chisel. Gun-shaped rongeur resected the right half of L4 lamina to the starting point of ligamentum flavum, removed ligamentum flavum, pulled the nerve root to the opposite side with a nerve retractor, exposed the right intervertebral space of L4, L5, and opened the window of sharp knife intervertebral disc. Nucleus pulposus and cartilage endplate were removed with nucleus pulposus forceps and scraper. Rinse the intervertebral space with normal saline, crush the lamina and put it into the intervertebral space, and then insert the interbody fusion cage filled with bone block until the position is suitable and the fluoroscopic position is satisfactory. The exploration and insertion of an enlarged spinal canal and nerve root canal to enlarge the spinal canal are satisfactory, and the nerve root is completely loosened.
3. Place the needle tip on the outer upper edge of the pedicle shadow. Under the monitoring of the C-arm machine, drill into the puncture needle, when the positive position shows that the puncture needle reaches the medial edge of the pedicle shadow, the lateral position shows that the needle tip reaches the posterior wall of the vertebral body, indicating that the safe puncture is completed, pull out the inner core of the puncture needle and place the human guide needle. Four pedicle screws were implanted into the pedicle along the guide needle. Then the guide needle was removed and the rod holder was implanted to connect the titanium rod and nut. Fluoroscopy showed that the reduction of the diseased vertebra was satisfactory.
4. After strictly counting gauze and instruments, alcohol disinfects the skin, and the wound is sutured with a single needle (Figure 2).

### Postoperative treatment

The postoperative treatment of the two groups was the same, and all patients were given symptomatic support treatment such as anti-inflammation and analgesia, nerve nutrition, and so on. Preoperative prevention and prophylactic use of antibiotics within 24 h after operation, patients were instructed to exercise properly in bed and ankle pump exercise to prevent the formation of deep venous thrombosis. Patients were encouraged to wear a waistline to get out of bed for daily activities on the second day after the operation. The dressing was changed every 3–5 days after the operation, and incision sutures were removed 14 days after the operation. Patients were advised to carry out rehabilitation exercises such as lumbar and dorsal muscles and lower limbs step by step.

### Observation indicators

The data from the two groups of patients were recorded: (1) operation time, radiation exposure time, intraoperative blood loss, bed rest time, hospitalization cost, and hospitalization time; (2) ODI score (The Oswestry Disability Index) (15), VAS score (Visual Analogue Scale) (16) of low back pain and lumbar vertebra, JOA (17) (Japanese Orthopaedic Association Scores) score 1 day before the operation; 1, 3, and 6 days after the operation; and 1, 3, and 6 months after the operation. The height, width, and intervertebral space height of intervertebral foramen were evaluated by imaging. Under the protection of waist support, patients are encouraged to get out of bed as soon as possible. The specific definition of observation indicators is as follows: JOA score, ODI score, and VAS score: evaluated by questionnaire at the corresponding time point. The VAS score was used for pain assessment. A line marked with 10 scales, marked 0 at the proximal end for no pain and 10 at the distal end for pain that cannot be recognized. When using it for the first time, teach the meaning of the scale to the patient, and show the back of the scale to the patient, ask it to mark the position that is consistent with the degree of pain, and the doctor will record it after converting it to the corresponding score according to the position. The higher the score, the more severe the pain. JOA score: mainly used to evaluate the function of lumbar vertebrae. It consists of four major items: subjective symptoms, limits of activities of daily living (Activity of Daily Living, ADL), clinical signs, and bladder function. The lowest score was 0 and the highest score was 29. The higher the score, the better the function. ODI score: a scale used to evaluate lumbar pain, function, and daily activity. It has high reliability and strong stability. It is mainly divided into 10 questions: the degree of pain, ADL ability, lifting ability, walking ability, sitting duration, standing duration, sleep status, sexual life, social participation ability, and outing ability. The total score is recorded according to the actual answer, that is, the actual score/5×(× represents the actual number of questions answered) × 100%. The higher the score, the higher the degree of obstacle. Operation time: the total time from skin incision to the completion of skin suture; intraoperative blood loss: the intraoperative blood loss was calculated indirectly according to the difference in hemoglobin before and after the operation. Postoperative bed rest time: from the first day after operation to the time when patients can get out of bed under the protection of lumbar support. In this study, we encouraged all patients in both groups to wear waistline to get out of bed on the second day after the operation, and record the time of getting out of bed early, walking 50 m at a time, about 10 min at a time, once or twice a day, and regard it as a sign of success in getting out of bed early. Hospitalization days refer to the total days from admission to discharge; intraoperative fluoroscopy time: C-arm x-ray machine automatically accumulates exposure time during operation.

### Statistical analysis

An independent *t*-test was used to compare the scores of VAS, JOA, and ODI before and during follow-up, and the operation time, hospital stay, intraoperative blood loss, and hospitalization cost were compared. The data were analyzed using the social science statistical software package Statistical Product and Service Solutions (SPSS) 26.0 (SPSS26.0, IBM, Armonk, NY, USA). *P *< 0.05 showed that there was a statistical difference.

## Results

### General data of the patients for the two groups

There was no significant difference in general data, complications, sex, age, and BMI between the two groups (*P *> 0.05). There was no significant difference in diagnostic constituent ratio between the two groups (*P *= 0.20), and there was no significant difference in fusion segment between the two groups (*P *= 0.77) (Table 1). A postoperative CT scan showed that no pedicle screw broke through the cortical bone of the medial and lateral walls of the pedicle. There were no complications such as nerve root, spinal cord, and vascular injury in both groups. The fusion cages were placed in the intervertebral space and did not enter the spinal canal or protrude anterior and lateral.

### Comparison of clinical effects between the two groups in perioperative period

There was no significant difference in bed rest time and hospital stay between the two groups (*P *> 0.05). The operation time in the Endo-LIF group was (203.35 ± 51.61) min, which was significantly longer than that in the MIS-TLIF group: (128.23 ± 27.68) min; the difference is statistically significant (*P *= 0.00). The postoperative hospital stay in the Endo-LIF group was 11.61 ± 2.85 days, which was significantly shorter than that in the MIS-TLIF group (*P *= 0.07). The difference in hemoglobin before and after operation in the Endo-LIF group was significantly lower than that in the MIS-TLIF group (*P *= 0.02). The intraoperative fluoroscopy time in the Endo-LIF group was significantly higher than that in the MIS-TLIF group (*P *= 0.00). The hospitalization cost in the Endo-LIF group was (69351.97 ± 630.03) RMB, which was significantly higher than that in the MIS-TLIF group: (62718.32 ± 1297.60) RMB; the difference is statistically significant (*P *= 0.00) (Table 2).

**Table 2:** Comparison of clinical outcomes between the two groups in perioperative period.

	Endo-LIF (31)	MIS-TLIF (31)	*P*
Duration of operation (min)	203.35 ± 51.61	128.23 ± 27.68	0.00
The difference of Hb before and after operation (g/L)	13.81 ± 2.13	20.61 ± 2.00	0.02
Time in bed (day)	3.45 ± 0.09	3.48 ± 0.09	0.80
Hospital stay (day)	11.61 ± 2.85	13.03 ± 3.21	0.07
Fluoroscopy time (s)	88.97 ± 0.26	73.55 ± 0.27	0.00
Hospitalization cost (RMB)	69,351.97 ± 630.03	62,718.32 ± 1,297.60	0.00

Endo-LIF, endoscopic lumbar interbody fusion; MIS-TLIF, minimally invasive surgery-transforaminal lumbar interbody fusion.

### Comparisons of ODI, VAS, and JOA scores

There was no significant difference in ODI score, JOA score, and VAS score between the two groups before operation (*P *> 0.05). There was no significant difference in ODI score, JOA score, and VAS score between the two groups at 1, 3, 6 days, 1, 3 months, and the last follow-up (*P *> 0.05). Intragroup comparison: there was a significant difference in VAS score before operation and last follow-up in the Endo-LIF group, ODI score before operation and last follow-up in Endo-LIF group, and JOA score before operation and last follow-up in Endo-LIF group (*P *= 0.00). There was a significant difference in VAS score before operation and the last follow-up in the MIS-TLIF group, ODI score before operation and last follow-up in the MIS-TLIF group, and JOA score before operation and last follow-up in the MIS-TLIF group (*P *= 0.00) (Table 3).

**Table 3:** Comparison of ODI, VAS, and JOA scores in the follow-up.

		Endo-LIF	MIS-TLIF	*P*
ODI	Before operation	87.90 ± 2.51	88.39 ± 2.38	0.44
	1 day after operation	64.35 ± 2.79	65.01 ± 5.48	0.56
	3 days after operation	61.35 ± 4.74	60.45 ± 7.94	0.59
	7 days after operation	50.48 ± 3.50	49.94 ± 1.21	0.41
	1 month after operation	44.52 ± 5.06	43.94 ± 2.48	0.57
	3 months after operation	24.19 ± 5.02	23.39 ± 2.38	0.42
	6 months after operation	13.10 ± 4.87	13.23 ± 4.75	0.91
VAS	Before operation	7.81 ± 0.75	8.13 ± 0.72	0.09
	1 day after operation	3.61 ± 0.50	3.45 ± 0.51	0.21
	3 days after operation	2.71 ± 0.46	2.65 ± 0.49	0.59
	7 days after operation	2.13 ± 0.62	2.26 ± 0.44	0.35
	1 month after operation	1.23 ± 0.43	1.16 ± 0.37	0.53
	3 months after operation	1.19 ± 0.40	1.10 ± 0.36	0.29
	6 months after operation	1.16 ± 0.37	1.10 ± 0.30	0.46
JOA	Before operation	9.61 ± 2.67	10.26 ± 2.39	0.32
	1 day after operation	20.61 ± 1.50	20.90 ± 1.47	0.44
	3 days after operation	21.90 ± 0.70	22.00 ± 0.63	0.57
	7 days after operation	23.23 ± 0.67	23.10 ± 0.65	0.43
	1 month after operation	25.61 ± 0.56	25.55 ± 0.51	0.64
	3 months after operation	26.77 ± 0.50	26.71 ± 0.46	0.60
	6 months after operation	27.71 ± 0.46	27.52 ± 0.51	0.12

Comparison of preoperative and last follow-up in the group, P < 0.05; Compare with Endo-LIF group and MIS-TLIF group.

ODI, The Oswestry Disability Index; VAS, Visual Analogue Scale; JOA, Japanese Orthopaedic Association score.; Endo-LIF, endoscopic lumbar interbody fusion; MIS-TLIF, minimally invasive surgery-transforaminal lumbar interbody fusion.

## Discussion

Lumbar degenerative disease is the most critical problem in the elderly. In the global population aging trend, lumbar degenerative disease is the most common cause of low back pain. In clinical practice, low back pain is a very common complaint about pain and disability among patients aged 65 years or above, and they are also the second most common age group for low back pain. Lumbar degenerative disease is caused by many factors, including but not limited to age, heredity, sex, obesity, physical activity, and occupation (such as repeated lifting), which is considered to change the natural process of lumbar facet joints and intervertebral discs leading to lumbar degenerative disease. Lumbar degenerative disease is a series of diseases, which may be characterized by disc herniation, scoliosis, spondylolysis, lumbar spondylolisthesis (18–21), spinal canal stenosis, and facet joint disease. Lumbar degenerative disease is a common health problem in middle-aged and elderly patients, which brings a heavy economic burden to individuals, families, and countries (22). For most patients with lumbar degenerative diseases, waist and leg pain can be relieved by traction, massage, or other conservative treatment. However, for patients whose conservative treatment is ineffective, surgical treatment should be considered. Spinal surgery plays an important role in nerve root pain, which can reduce the pain and disability of patients. The surgical treatment of patients with lumbar degenerative diseases can be divided into traditional open surgery and minimally invasive spinal surgery. The key to lumbar fusion surgery is the implantation of an interbody fusion cage, with the passage of time, the technology of interbody surgery is also improving. Traditional open surgery translaminar lumbar interbody fusion (PLIF) will cause greater damage to the posterior structure of the spine, resulting in lumbar instability, muscle injury, epidural scar adhesion, and other complications, which is the most important problem after the operation, affecting the surgical effect (23). Over the past few decades, due to the renewal and progress of surgical instruments, as well as the progress and innovation of minimally invasive spinal surgery technology, the minimally invasive road of spinal surgery has gone further and further.

**Figure 3:**
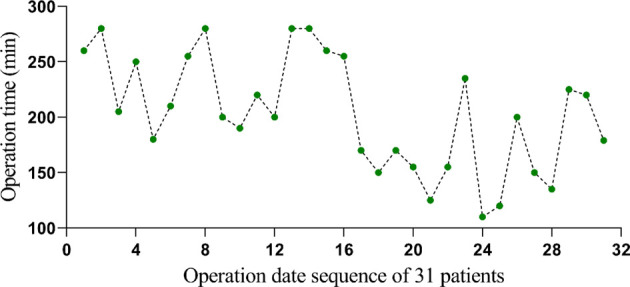
Change chart of Endo-LIF operation time.

Endo-LIF and MIS-TLIF are safe and effective minimally invasive surgical techniques for the treatment of lumbar disc herniation. MIS-TLIF, first described by Foley et al., is a transforaminal lumbar interbody fusion characterized by the insertion of a tubular retractor through muscle dilation exposure to reduce approach-related complications. MIS-TLIF is a safe and effective minimally invasive technique for the treatment of various lumbar degenerative diseases, including primary degenerative lumbar disease in one or more lumbar segments. It can directly decompress the ipsilateral nerve root. Therefore, severe low back pain caused by degenerative lumbar diseases, instability of intervertebral segments, instability after laminectomy, multiple recurrent disc herniation, spinal trauma, intervertebral foramen stenosis with deformity, and degenerative scoliosis are all potential indications for MIS-TLIF. Usually grade I or II spondylolisthesis is also an indication of MIS-TLIF surgery, which causes mechanical low back pain or nerve root pain. Open posterior surgery may be a better option for patients with highly severe spondylolisthesis, and MIS-TLIF surgery is technically challenging for most spinal surgeons. Most importantly, in some patients, there are conjoined nerve roots in the lumbar intervertebral foramen, which is one of the contraindications of MIS-TLIF. For these multisegmental patients who need MIS-TLIF surgery, lumbar magnetic resonance imaging should be examined carefully before operation. In recent years, spinal endoscope has been widely used in the treatment of degenerative lumbar diseases. MIS-TLIF has become an acceptable and popular lumbar fusion technique in spinal surgery. In recent years, many literature studies have compared the therapeutic effects of MIS-TLIF and traditional TLIF and confirmed that MIS-TLIF can achieve the same effect as open surgery. Percutaneous intervertebral foramen endoscopy is the most commonly used spinal endoscopic system at present. Its basic operation is to puncture the intervertebral foramen through the posterolateral side, dilate the intervertebral foramen step by step, enlarge the intervertebral foramen, implant the passage and complete the nerve decompression operation under an endoscope. Percutaneous intervertebral foramen endoscopy is the most minimally invasive intervertebral disc surgery system by percutaneous puncture and entering through the natural space of the safe triangle. More than 10 years ago, after the intervertebral foramen mirror entered China, it opened the second revolution of minimally invasive spinal surgery technology. Because the percutaneous intervertebral foramen endoscopy technology is very minimally invasive, and its surgical indications are constantly expanding, so it has been loved by patients and doctors. Percutaneous intervertebral foramen endoscopy has witnessed the overall development of our minimally invasive spinal surgery technology. In 1983, Kambin first reported lumbar discectomy assisted by posterolateral arthroscopy. Because the operation could not be performed visually at that time, countermeasure catheterization and contralateral arthroscopy were used, but this method placed a passage on the normal side, which made the operation tedious and increased the risk of contralateral lumbar disc and nerve injury. On this basis, total endoscopic spinal disc technology arises at a historic moment. In 1997, Yeung put forward YESS technology, that is, the third generation of spinal endoscopic YESS system, which combines spinal endoscopy with working channels. In 2003, Hoogland proposed the TESSYS technology and system, which includes foramen plasty and intervertebral disc operation. In 1990, Kambin described the safe transforaminal triangle, emphasizing the optimal decompression of nerve structures within the intervertebral disc and under endoscopy. The Kambin triangle provides us with a safe area for us to enter the intervertebral disc with a different area for everyone and different lumbar segments with different sizes. What is more important is that the Kambin triangle is not constant. The Kambin triangle will change in size according to the doctor's puncture angle and direction. Lumbar degeneration, such as hypertrophy of the articular process, also affects the area of Kambin's triangle. The Endo-LIF technique is a perfect combination of minimally invasive decompression and fusion with percutaneous intervertebral foramen endoscopy, which is usually used in surgical patients with single segmental degeneration or spinal fusion. Endo-LIF is characterized by small trauma, little bleeding, good intraoperative field of view, low risk of nerve injury, and sufficient amount of intervertebral bone grafting (13). At present, the complete Endo-LIF has not been reported in the current study; there are still few studies on its indications and clinical efficacy, and there are few reports on the comparison of its efficacy with MIS-TLIF. In this study, a strict prospective clinical randomized controlled design was conducted to compare the difference in short-term clinical efficacy between the two methods. In this study, there was no significant difference in general data, complications, sex, age, and BMI between the two groups. There was no significant difference in diagnostic constituent ratio between the two groups (pause 0.204), and there was no significant difference in fusion segment between the two groups (pause 0.768). There was no significant difference in ODI score, JOA score, and VAS score between the two groups before operation. There was no significant difference in ODI score, JOA score, and VAS score between the two groups at 1, 3, 6 days, 1, 3 months, and the last follow-up. It may be because the nail placement (24) in the two groups is the same, so the trauma caused by nail placement is the same. The main difference between the two groups was the implantation of the fusion cage. Both MIS-TLIF and Endo-LIF belong to minimally invasive spinal surgery. MIS-TLIF surgery is through the placement of expandable channels, can maximize the protection of muscles, to avoid extensive muscle peeling, the disadvantage of this operation is that there is a certain pressure on the surrounding muscles, and in order to ensure a clear field of vision, it is necessary to constantly deal with the muscles slipping out of the gap and bottom of the expansion plate (usually removed by direct electric knife). In Endo-LIF operation, the channel of implant fusion is smaller than that of MIS-TLIF operation, and the incision is smaller, so it does not need to squeeze the surrounding muscle tissue, only needs to be treated under a microscope, and does not need to deal with the problem of slipping out of the muscle. So in theory, the trauma of Endo-LIF surgery should be less than that of MIS-TLIF surgery, but from the clinical observation, this slight difference is not reflected in these pain and function scores. In the implantation of the fusion cage, both methods are the same, both are TLIF, and the bone range is the same, including the inferior joint and part of the superior articular process as well as the endplate. There is no significant difference between the two groups, which may have something to do with the small sample size, and then continue to increase the sample size to obtain different results. At the last follow-up, the fusion rate was 100% in the MIS-TLIF group and 100% in the Endo-LIF group. There was no difference in the fusion effect between the two groups. Perhaps because the two groups of surgical patients are using a large number of autogenous bones, in the endplate treatment, MIS-TLIF surgery is semi-open, direct treatment, and Endo-LIF group is through the placement of channels, blindness, and microscopic treatment; both can effectively deal with the endplate. A patient who underwent Endo-LIF surgery found that the fusion cage shifted backward 1 month after the operation, and the patient had no symptoms of discomfort. The patient was instructed to stay in bed for 2 months, and the patient recovered well 3 months after the operation. The reason for the displacement of the fusion cage may be related to the large amount of activity and range of movement of the patients after the operation. The time of percutaneous nail placement in the two groups is the same, and the operation time in the MIS-TLIF group is shorter, probably because the implantation of the fusion cage is different from that of Endo-LIF: the establishment of surgical passage is easier and saves time; the efficiency of dealing with the bony spinal canal is high, and open tools including ultrasonic bone knife can be used; endplate processing is fast, similar to lumbar open surgery. The Endo-LIF group takes a long time to open the bony spinal canal, but currently uses a ring saw, the combination of fluoroscopy and microscope, lack of other more effective methods, and the efficiency are different from that of the MIS-TLIF group; the endplate treatment needs the combination of blind vision under channel protection and microscope, so it takes a long time. The more times of fluoroscopy in the Endo-LIF group is also one of the reasons that affect the operation time: at the level of the facet joint treated in the first ring, fluoroscopic monitoring is needed to control the depth of the ring saw and prevent it from protruding into the spinal canal, resulting in nerve injury. In the stage of implanting the fusion cage, more fluoroscopic monitoring is needed to complete, which increases the number of fluoroscopies and increases the operation time. Most doctors who have completed Endo-LIF surgery are still in the initial stage of the learning curve (25) and the operation time is long. From this study, it can be found that the operation time of Endo-LIF surgery becomes shorter with the passage of time (Figure 3). So as the technology becomes more skilled, the gap will narrow. Because continuous irrigation of normal saline under an endoscope is needed during the Endo-LIF operation, it is impossible to accurately calculate the intraoperative blood loss. By comparing the difference in hemoglobin before and after operation between the two groups, and indirectly comparing the amount of intraoperative blood loss, the difference in hemoglobin before and after operation in the Endo-LIF group is (13.81 ± 2.13) g, and the difference before and after the operation is (20.61 ± 2.00) g/L. There was a significant difference in the amount of intraoperative blood loss between the two groups (pause 0.02). Endo-LIF surgery has less intraoperative bleeding and has more advantages; on the one hand, it may be because Endo-LIF surgery requires less soft tissue treatment than MIS-TLIF surgery and, on the other hand, it may be because there is the certain water pressure in Endo-LIF surgery, so it can reduce intraoperative bleeding, open the spinal canal during the operation, and reduce the bleeding of intraspinal venous plexus. In addition to certain water pressure, the fusion field under the microscope is better, which can enlarge the visual field. The vascular plexus can be seen clearly, and the radiofrequency knife head under the microscope can accurately stop the bleeding, but the use of a radiofrequency head also increases the hospitalization cost, which makes the hospitalization cost of the Endo-LIF group higher. There are still some shortcomings in this study. First of all, the sample size of this study is small, and the follow-up time is short, so the safety and effectiveness of the operation cannot be fully evaluated. Second, the surgical indications are limited, and it is still a contraindication for patients with lumbar spondylolisthesis of degree II or above. In the future, multicenter, large sample, and long-term follow-up studies are needed to further verify the clinical efficacy of percutaneous endoscopic-assisted transforaminal lumbar decompression and fusion (Endo-LIF).

## Conclusions

According to the evidence provided by our study, there is no significant difference in short-term clinical efficacy and safety between Endo-LIF and MIS-TLIF in the treatment of single-segment degenerative lumbar diseases, but MIS-TLIF surgery is significantly better than Endo-LIF surgery in operation time, hospitalization cost, and fluoroscopy time, and Endo-LIF surgery are significantly better than MIS-TLIF surgery in terms of intraoperative blood loss. The clinical efficacy and success rate of percutaneous endoscope-assisted transforaminal lumbar decompression and fusion (Endo-LIF) and minimally invasive transforaminal lumbar decompression and fusion (MIS-TLIF) meet the clinical requirements and have application prospects.

## Data Availability

The original contributions presented in the study are included in the article/Supplementary Material, further inquiries can be directed to the corresponding author.
